# Transgenic animal models to explore and modulate the blood brain and blood retinal barriers of the CNS

**DOI:** 10.1186/s12987-022-00386-0

**Published:** 2022-11-01

**Authors:** Andreia Goncalves, David A. Antonetti

**Affiliations:** Department of Ophthalmology and Visual Sciences, University of Michigan Kellogg Eye Center, 1000 Wall St Rm, Ann Arbor, MI 7317 USA

**Keywords:** Blood–brain barrier, Blood-retinal barrier, Transgenic animal models, Neurovascular unit, Central nervous system disorders, Tight junctions

## Abstract

The unique environment of the brain and retina is tightly regulated by blood–brain barrier and the blood-retinal barrier, respectively, to ensure proper neuronal function. Endothelial cells within these tissues possess distinct properties that allow for controlled passage of solutes and fluids. Pericytes, glia cells and neurons signal to endothelial cells (ECs) to form and maintain the barriers and control blood flow, helping to create the neurovascular unit. This barrier is lost in a wide range of diseases affecting the central nervous system (CNS) and retina such as brain tumors, stroke, dementia, and in the eye, diabetic retinopathy, retinal vein occlusions and age-related macular degeneration to name prominent examples. Recent studies directly link barrier changes to promotion of disease pathology and degradation of neuronal function. Understanding how these barriers form and how to restore these barriers in disease provides an important point for therapeutic intervention. This review aims to describe the fundamentals of the blood-tissue barriers of the CNS and how the use of transgenic animal models led to our current understanding of the molecular framework of these barriers. The review also highlights examples of targeting barrier properties to protect neuronal function in disease states.

## Introduction

The central nervous system (CNS), including the brain and retina, has a relatively high energy requirement due to neuronal activity, but in order to maintain proper neuronal function, the neural micro-environment must be rigorously controlled. These organs are highly vascularized and in the case of the retina, possess a unique vasculature structure, that provide the necessary oxygen and glucose for proper neuronal activity. To safeguard the neuronal environment, the vessels in the CNS possess well developed junctional complexes in the vascular endothelium, contributing to the formation of the blood–brain (BBB) or inner blood-retinal barrier (iBRB) controlling paracellular flux [1, 2]. In combination with active transcellular transporters, the BBB and BRB act as selective filters that regulate the flux of fluids, signaling molecules, metabolic intermediates, and cells into and out of the CNS, regulating the neural environment and distinguishing the microvasculature of the brain and retina from that of other vascular beds. The development of BBB/BRB, or barriergenesis, occurs spatially and temporally along with angiogenesis in the CNS [3]. In a wide variety of disease affecting proper neural function in the CNS including stroke, brain tumors, dementia and in the retina, diabetes, retinal vein occlusions and age-related macular degeneration, loss of the BBB and BRB contribute to disease pathogenesis [4, 5].

This review will focus on the vascular components creating the BBB and BRB although both the brain and retina possess additional membranes necessary for proper control of fluids and solute movement to the CNS. These include the choroid plexus with an epithelial layer that filters fluid generating cerebrospinal fluid (CSF) and constitutes the blood-CSF barrier [6] and the retinal pigmented epithelium adjacent to photoreceptors creating the outer BRB [7].

**Fig. 1:**
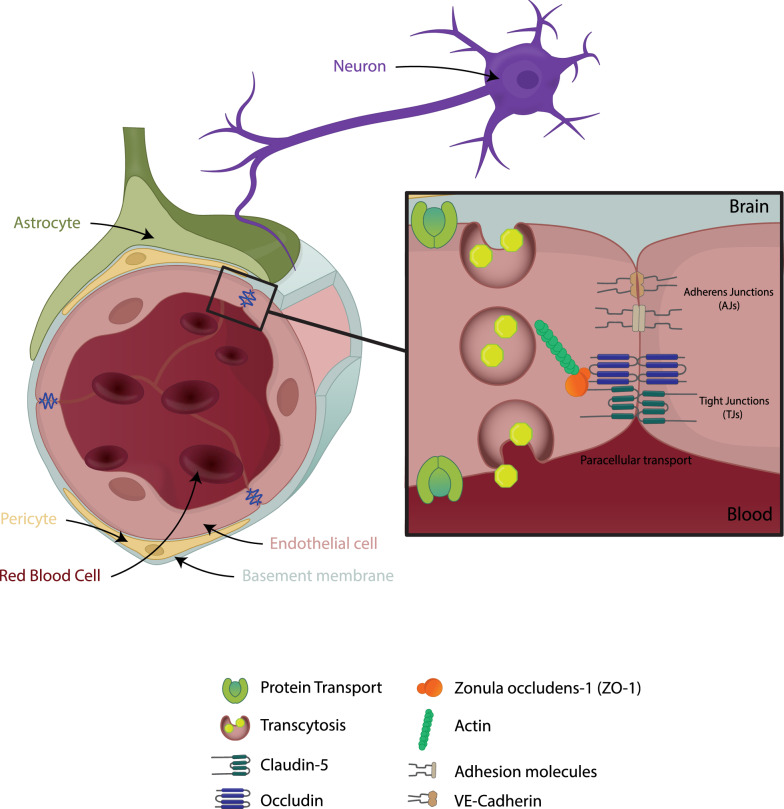
The Neurovascular Unit. In the CNS, ECs, pericytes, glia and neurons form an interdependency that allows for proper neuronal function and establishes a blood-tissue barrier that controls the passage of solutes and fluids. The BBB and BRB ECs possess unique characteristics that allow for this control, specifically the presence of TJs that seal the paracellular space and low rates of transcytosis. TJs are formed by long strands of transmembrane proteins including: claudins, occludin and tricellulin that are connected to the actin cytoskeleton by scaffold ZO proteins

Brain and retina ECs possess certain properties that are unique and contribute to the integrity of the BBB and BRB [2]. They have continuous intercellular tight junctions (TJs) that control the passage of ions and nutrients within the paracellular space. They have limited vesicles reducing the rate of transcytosis, creating a transcellular barrier to hydrophilic molecules. Finally, and as a consequence of their limited permeability, the expression of dedicated transporters facilitates nutrient uptake such as glucose, amino acids, and metabolically relevant ions and removal of potentially neurotoxic substances and drugs. These properties of the BBB have been reviewed in detail in [2] and are depicted in Fig. 1. A recent study has shown that neural activity regulates endothelial barrier function specifically related to efflux transporters [8]. Using a transgenic approach to stimulate or inhibit glutamatergic neurons through designer receptors exclusively activated by designer drugs (DREADDs) the investigators demonstrated that BBB efflux, particularly through the transporter P-glycoprotein, is regulated by neuronal activity through endothelial circadian clock genes [8].

Animal models have been indispensable in clarifying the underlying mechanisms of controlled transport, barrier permeability, cellular architecture, and related morphological changes, which have provided most of our basic knowledge about the BBB/BRB. Recent research utilizing transgenic animals has identified critical elements in the structural components of the BBB and BRB as well as elucidate signaling factors required for the developmental process that controls the formation of the BBB and BRB in the CNS. Furthermore, studies have begun to elucidate how changes to the BBB and BRB affect CNS function and contribute to disease pathogenesis and have begun to explore potential mechanisms to prevent loss of BBB and BRB or restore these barriers in order to intervene in disease processes. This review will specifically explore these transgenic mouse models used to study the development and functional significance of the BBB and BRB in normal physiology and disease focusing on what transgenic models have revealed about the protein components that make up the BBB and BRB and required pathways in barriergenesis. Finally, this review will highlight studies that elucidate the relationship of the BBB and BRB to proper neural function and the neurovascular unit.

### Transport across the BBB/BRB

#### Tight junctions and paracellular transport

At the BBB and iBRB, signals from glial cells and pericytes induce vascular ECs to form well-developed TJs that restrict the passage of molecules through the paracellular space thus sealing the blood vessel lumen from the CNS parenchyma [9]. Additionally, TJs also have roles in numerous signaling pathways involved in their assembly, function, and polarity as well as a role in gene expression [10, 11].

The TJ complex consists of numerous interacting proteins and include transmembrane claudin family members, MARVEL (MAL and related proteins for vesicle trafficking and membrane link) proteins, and junctional adhesion molecules (JAMs), which form intercellular contacts (Fig. 1) [10]. These transmembrane proteins are linked to the actin cytoskeleton by cytoplasmic scaffold proteins including zonula occludens proteins (ZO-1, ZO-2 and ZO-3) [2]. Recent studies using gene deletion and conditional gene deletion have shed important insight into the proteins that regulate the paracellular permeability in the BBB and BRB.

#### Claudin family

The claudin family is considered the main structural component of the TJs, forming the intramembrane strands. They have a central role in controlling paracellular transport by forming charge specific interactions between claudins that restrict or allow particular ion diffusion and restrict solute permeability [12]. At the plasma membrane, claudins interact with each other across opposing cells (trans-interaction), with the interaction between the same claudin type being a homotypic interaction and between different claudins a heterotypic interaction [13, 14]. In addition, claudins interact within the same cell (cis-interaction) in a homomeric or heteromeric interaction [13, 14]. Structurally, the extracellular loops (ECL) of claudins determine their functionality, with the first ECL being involved in the ion selectivity of claudin strands while the second ECL seems to be important for the intercellular binding that narrows the paracellular space [14, 15]. Additionally, almost every claudin (except for claudin-12) has a PDZ binding motif at the C-terminal that allows binding to the PDZ domain of cytoplasmic scaffolding proteins such as ZO-1, determining its spatial organization [16].

The claudin family is composed of 27 members [17] and they can functionally be divided into barrier sealing or pore forming claudins. Examples of sealing claudins include claudin-1, 3 and 5 that are found in several epithelia and endothelia with a moderate to high transelectrical resistance [18, 19]; while example of pore-forming claudins include claudin-2, -10, -16, -17 and -19 [20] that allow charge and size restricted permeability. The expression and combination of specific claudin members determines size and charge-selectivity and permeability of the TJ in a tissue specific manner. During development and in response to disease stress, claudin expression patterns change modulating paracellular permeability [10, 21].

Expression and localization of claudins-1, -3, -5, and -12 at the BBB and BRB have been reported, where claudin-5 seems to be the most highly enriched [22–24]. However, the lack of specific claudin antibodies has led to erroneous reports regarding which claudins are expressed at the BBB and BRB. The generation of more recent knockout mice and single-cell RNA sequencing technique have allowed more direct assessment of the contribution of claudins to the BBB and BRB. Claudin-1 knockout mice die within 1 day after birth due to excessive dehydration from the skin demonstrating that claudin-1 has a key role in skin barrier formation [25]. No BBB defects were reported which has led to the suggestion that claudin-1 is not essential for BBB TJ complex function under physiological conditions. More recent data has suggested claudin-1 expression is rather associated with altered TJ function in pathology, such as ischemic stroke delaying barrier reformation [26]. Claudin-3 was thought to be enriched at the BBB; however, a recent study failed to identify claudin-3 expression in brain microvessels from wildtype animals by single cell RNA sequencing or microvessel isolation and qRT-PCR [27]. Further, claudin-3 knockout mice demonstrated that absence of claudin-3 does not impair brain barrier function [27]. A separate study showed that claudin-3 knockout mice did present with exacerbated ischemic lesion in a mouse model of stroke resulting in BBB breakdown associated with a decrease in claudin-1 expression and shorter TJ strands [28]. Claudin-12 knockout mice were recently generated and revealed that this claudin is not essential in establishing or maintaining BBB TJs [29]. Additionally, using a claudin-12 reporter mouse it was shown that this claudin has a very low expression in BBB ECs, being predominantly expressed in neurons, astrocytes and smooth muscle cells [22, 29]. Whether other claudins compensate after gene deletion of a particular claudin remains a possibility in these types of studies. But current evidence from single cell sequencing, PCR or gene marker studies have revealed under normal conditions claudins (1, 3 and 12) are either not expressed or expressed at low concentration in brain vascular endothelium and their loss by gene deletion does not alter barrier properties revealing these claudins are not necessary for the BBB.

Claudin-5 remains the one claudin clearly present and required for BBB. The importance of claudin-5 at the BBB was demonstrated by Nitta et al., revealing that claudin-5 knockout mice died shortly after birth due to loss of size-selectivity permeability of the BBB, allowing the diffusion of molecules smaller than 800 Da, but not of larger molecules [30]. Surprisingly, these mice showed no abnormal vessel development or edema, nor did they present morphologic alterations in TJ strands. Whether other claudins or TJ proteins compensate for the lack of claudin-5 in TJ formation remains unknown. Claudin-5 also seems to be the highest expressed claudin at the BRB [31]. However, since claudin-5 knockout mice die before the retinal vasculature is formed its direct role in BRB permeability has yet to be explored. Nonetheless, decreased claudin-5 expression correlates with increased BBB and BRB permeability in several models of disease [32].

#### MARVEL and angulin family

The TJ associated MARVEL proteins (TAMP) family includes occludin (MARVELD1), tricellulin (MARVELD2) and MARVELD3. Similar to the claudin family, they have 4 transmembrane domains with 2 ECL domains, however they cannot form TJ strands alone [33]. They are thought to enhance strength of claudin strands through cis interactions [34].

Occludin was the first transmembrane TJ protein to be described [35] and its high expression at the BBB and BRB may be a crucial determinant of the TJ permeability properties of these endothelial cell when compared to other vascular beds [36]. Surprisingly, occludin knockout mice revealed that occludin was not required for TJ strand formation as these animals presented intact BBB with morphologically normal TJs [37]. These mice exhibit complex phenotypes with hyperplasia of the gastric epithelium, brain calcification, thinning of the compact bone and male infertility [37, 38]. However, this occludin knockout mouse, thought to be a homozygous null model, still expressed a recently described occludin isoform (Ocln-ΔN) that lacks the N-terminus and the first 3 transmembrane domains [39]. Since this isoform still contains the C-terminus, which has been shown to mediate ZO interactions, localization to the TJ and essential signaling [40, 41], the definite contribution of occludin in BBB formation remains unknown. The role of occludin in regulating barrier properties and maintaining paracellular permeability has become clearer with recent research. Phosphorylation of several residues in the C-terminus region have been shown to regulate occludin interaction with ZO proteins and TJ integrity [42–45].

Tricellulin (MARVELD2) is another transmembrane protein that is present specifically in tricellular endothelial junctions in the brain and retina [46, 47]. Several studies have shown that ECs of CNS are particularly enriched with tricellulin when compared to other vascular beds [22, 23, 48]. Even though tricellulin and occludin only share 32% homology, tricellulin has been shown to compensate for the lack of occludin, relocating to bicellular TJs [46, 49], suggesting a compensatory mechanism. Additionally, mice lacking tricellulin are also viable but develop hearing loss, similar to occludin deficient mice [49–51]. Recently, it has been shown that tricellulin and tricellular junctions might be involved in regulating T cell entry into the CNS [48]. Furthermore, using Madine Darby Canine Kidney cells, a clear role for TAMP family members occludin and tricellulin was demonstrated by creating double knockout lines [52]. In these experiments, loss of occludin or tricellulin alone had limited effect on barrier properties. However, in the double knockout lines, electrical resistance was reduced in half and small solute flux increased fivefold associated with loss of TJ branching, observed by electron microscopy. These studies support a role for Marvel D1 and 2 having a specific role in TJ branch formation, increasing TJ complexity and strengthening barrier function. The role for occludin and tricellulin gene deletion on the BBB has yet to be established.

Lipolysis-stimulated lipoprotein receptor (LSR) (also known as angulin-1), has been shown to be necessary to recruit tricellulin to tricellular junctions [53]. However, despite limited tricellular junctions in the BBB vascular compared to epithelial monolayers, LSR has been found to be specifically expressed at the BBB and BRB [47, 54]. LSR is a type I trans-membrane proteins with an extracellular immunoglobulin-like domain. The intracellular domain is responsible for the recruitment of tricellulin to the tricellular TJ [55]. And the extracellular domain is thought to form a homophilic trimer structure at tricellular contacts [56]. LSR knockout are embryonic lethal [57] as the BBB fails to seal with a size-selective permeability defect to small molecules [54]. However, these mice do not present any ultrastructural differences in TJ strands, resembling the claudin-5 knockout [30]. These studies point to LSR as having a central role in tricellular TJ assembly and BBB barriergenesis.

MARVELD3 is the third member of the TAMP family and is also a tetraspan protein; however, it lacks the C-terminus found in occludin and tricellulin [58] and its role at the BBB/BRB remains unknown. TJs are known to regulate signaling mechanisms, and MARVELD3 was shown to be a dynamic junctional regulator of the MEKK1–c-Jun NH_2_-terminal kinase (JNK) pathway. Loss of MarvelD3 expression in epithelial cells resulted in increased cell migration and proliferation [59]. MARVELD3 was also identified as a regulator of ocular morphogenesis, as its depletion disrupted the normal expression pattern of eye-field transcription factors and led to the development of smaller eyes or to an eye-less phenotype [60].

#### JAMs

JAMs are a family of single span proteins with an extracellular immunoglobulin-like domain [61]. They interact with ZO-1 through a PDZ domain at the C-terminus and can dimerize through both cis*-* or trans-interaction [62, 63]. Dimerization of JAM-A is required for its interaction with the scaffolding proteins and TJs assembly [64, 65].

In brain and retinal ECs, JAM-A is the predominant JAM isoform [66, 67]. JAM family members have been shown to also regulate leukocyte adherence and transmigration by interacting with integrins expressed on leukocytes [68], endothelial cell-specific deletion of JAM-A blocked leukocyte migration through venular walls [69]. JAM-C deficient animals showed increased JAM-A expression and enhanced retinal vascularization [70, 71], suggesting that they have compensatory roles and denote a role for JAM-A in retinal angiogenesis.

#### Zonula occludens

Zonula occludens (ZO) are part of the MAGUK family and act as scaffold proteins that link transmembrane proteins to the actin cytoskeleton stabilizing TJ strands [72]. Three isoforms have been described, ZO-1, -2 and -3 [73–75]. ZO-1 and ZO-2, but not ZO-3, bind the transmembrane TJ proteins to the actin cytoskeleton at the BBB, allowing for TJ complexes to assemble [76]. At the structural levels, PDZ domains allow ZO-1 to interact with other ZO, claudins and JAMs [16, 40, 77] while the SH3-GUK domains together allow occludin interaction [41]. In addition, several cell signaling molecules and additional junction proteins and cytoskeletal proteins bind to and are organized by ZO family members [78].

The central role of ZO family in the formation of TJs has been demonstrated in cells deficient for ZO-1 and -2 which fail to form TJs with impaired claudin strand polymerization [79, 80]. ZO-1 knockout mice are embryonic lethal showing vascular defects with abnormal angiogenesis in the yolk sac [81]. ZO-2 deficient mice are also embryonic lethal, with embryos dying shortly after implantation due to increased gastrula apoptosis [82], suggesting that these proteins do not have redundant functions in early development. ZO-1 and -2 also have a role in regulating cell proliferation through binding and regulating localization of the transcription factor ZO-1 associated nucleic acid binding protein (ZONAB) [83]. ZO-1 has also been shown to regulate cell–cell tension, cell migration and angiogenesis [84].

#### Transcellular transport and caveolae

In ECs of the CNS small lipophilic molecules can passively diffuse across the lipid bilayer but, in many cases, are returned to the blood by efflux transporters. Larger lipophilic molecules along with hydrophilic molecules and ions are dependent on transcellular transport to cross the BBB/BRB through channels, pinocytosis, receptor and non-receptor mediated vesicular transport and excluded by efflux pumps [85, 86]. To maintain tight control of neuronal environment of the CNS, ECs have low rates of transcytosis, express low levels of transporters while expressing high levels of efflux pumps.

Some transporters are expressed early in development, such as the glucose transporter GLUT1, and its distribution pattern correlates with BBB maturation [87]. GLUT-1 haploinsufficient mice have severe defects in BBB physiology and neurovascular function, showing decreased brain glucose uptake, reduced cerebral blood flow and increased BBB permeability due to lower TJ protein expression [88, 89]. GLUT-1 deletion in ECs not only affects glucose uptake to the brain but may also alter endothelial metabolism contributing to cell dysfunction or death [90]. This suggests that loss of BBB specific transporters can ultimately influence general BBB integrity.

Transcytosis in ECs occurs primarily through caveola. Caveolae are plasma membrane invaginations consisting of lipid rafts and the proteins caveolin-1 or flotillin-1 and cavin. Caveolae vesicles undergo endocytosis and within the cytoplasm of cells can initiate signal transduction, be recycled to the plasma membrane, or transport their contents to the opposite plasma membrane [91]. Caveolin-1 knockout mice have a significant increase in BRB permeability, potentially due to compensatory flux through the paracellular pathway, specifically in branch veins, without apparent alteration in junctional proteins [92]. Plasmalemma vesicle-associated protein (PLVAP), a structural protein of caveolae in peripheral ECs is absent or has a low expression in BBB/BRB ECs [93]. However, PLVAP is up-regulated in a number of diseases characterized by increased BBB/BRB leakage and is spatially correlating with increased vascular permeability sites [94–96], making PLVAP a good marker for increased transcytosis specifically in CNS EC. Data suggests that during barriergenesis, transcellular and paracellular control of permeability are coordinated at the BRB with high permeability vessels possessing both high PLVAP and low claudin-5 expressing ECs [97]. In the brain, a similar mechanism is found, where circumventricular organs vessels, which physiologically lack BBB properties, have high PLVAP and low claudin-5 expressing ECs as opposed to the tight BBB vessels that have low PLVAP and high claudin-5 expressing ECs [98].

Another molecule implicated in regulating transcytosis in the CNS is the transporter major facilitator superfamily domain-containing 2a (Mfsd2a). In brain ECs, Mfsd2a transports phospholipids from the outer to the inner leaflet of the plasma membrane, including omega-3 fatty acids like docosahexaenoic acid (DHA), which is important for brain development [99]. Gene deletion studies revealed that lack of Mfsd2a leads to microcephaly, neuronal cell loss and cognitive deficits [100, 101]. These mice were also shown to have increased BBB and BRB permeability due to increased levels of transcytosis without TJ alterations [100, 102]. In contrast, other studies have failed to detect BRB breakdown in Mfsd2a deficient mice using the same molecular weight tracers [103, 104]. Besides ECs, Mfsd2a is also expressed in astrocytes which may lead to insight to resolve the contradicting studies [22]. Nonetheless, these studies did show that DHA transport by Mfsd2a was essential for long-term photoreceptor survival, denoting the importance the lipid transport by Mfsd2a in retinal homeostasis [103, 104]. Subsequent work using endothelial-specific transgenic mice with a mutation in Mfsd2a that blocks its transporter activity (Mfsd2a D96A) revealed that the increase in unsaturated phospholipids by functional Mfsd2a alters the lipid content of the plasma membrane, which inhibits the development of caveolae and hence prevents transcytosis across the BBB [105], contributing to BBB formation and maturation.

### Neurovascular unit

The development and maintenance of the BBB and BRB results from the close contact of the vasculature with neurons, glial cells and pericytes and may also include microglia [106]. The term neurovascular unit (NVU) is used to describe the interdependence of these cells for proper neural signaling. The vasculature barrier and control of blood flow ensure proper neural support and maintain the required environment for neuronal function, while neurons, glia and pericytes signal to ECs to induce and maintain the unique barrier properties of the BBB and BRB and control proper blood flow (Fig. 1). Early evidence for the importance and requirement of such signaling was demonstrated through studies showing that transplantation of CNS neural tissue to the periphery can drive ectopic BBB formation [107, 108]. More recent data using transgenic mice to specifically elucidate the signaling mechanisms of BBB and BRB formation are highlighted below.

#### Pericytes

Pericytes are mural cells that ensheathe the abluminal surfaces of CNS capillaries and support microvessel stability and regulation of blood flow [109, 110]. In contrast with other vascular beds the ratio of pericytes to ECs is significantly higher in the CNS, with 1 pericyte:1 endothelial cell in the retina [111] and 1 pericyte: 4 endothelial cells in the brain [112], suggesting that pericytes play an important role in the BBB and BRB.

Pericytes are recruited to the CNS vasculature by release of platelet-derived growth factor-b (Pdgf-b) from nascent ECs during embryonic development, which binds to platelet-derived growth factor receptor-β (Pdgfrβ) expressed on the pericyte cell surface. This recruitment signaling was demonstrated in both Pdgfb and Pdgfrb knockout mice which completely lack CNS pericytes [113, 114]. Pericyte-deficient mice are embryonically lethal and are characterized by high vascular permeability, showing increased transcytosis as well as TJ defects, demonstrating that pericytes are required for BBB formation [115–117]. Additionally, decreasing pericyte recruitment without completely abolishing it revealed that adult mice present a strong correlation between pericyte density and BBB permeability [116, 117]. However, Park and colleagues recently showed that pericyte ablation in the adult mouse using conditionally expressed diphtheria toxin did not lead to BRB leakiness, suggesting that pericytes are important for initial BBB formation but not maintenance [118]. The same study also demonstrated that loss of pericytes made the retinal vessels highly susceptible to VEGF signaling, leading to increased hemorrhage and vascular permeability, suggesting that pathological pericyte loss may contribute to barrier loss and disease etiology.

In general ECs and pericytes are separated by a shared extracellular matrix (ECM); however, in certain areas, they form specialized junctions called peg and socket which allows for direct signaling between pericytes and ECs [119]. After recruitment, adhesion between ECs and pericytes is mediated by transforming growth factor-β (TGF-β) [120]. Both cell types secrete TGF-β and express its receptor TGF-βR2, which activates Smad pathway to promote transcription of target genes. TGF-β signaling promotes the production of ECM molecules by pericytes and upregulates N-cadherin, an adherens junction protein, in endothelial cells enhancing pericyte adhesion [120]. Endothelial specific deletion of Smad4 induces pericyte detachment, vascular defects, increased BBB permeability, and hemorrhage [121]. Pericytes also promote the known Ang-Tie2 angiogenic pathway by secreting Ang 1 and Ang2 that signal to Tie2 receptor in ECs. While Ang1 is known to stabilize vessels via Tie2 receptor in ECs [122], its counterpart, Ang2 can act as an antagonist of Ang1 signaling to Tie2 leading to ECs destabilization and retinal overexpression of Ang2 in mice leads to pericyte detachment and increased BRB leakage [123]. Studies using intravitreal injection of Ang2 revealed that the loss of endothelial barrier function was associated with induction of VE-Cadherin phosphorylation and degradation [124]. At the BBB, endothelial cell-specific expression of Ang2 gain-of-function was shown to increase vascular permeability via an increase in both paracellular and transcellular routes along with reduced pericyte coverage [125]. Ang2 gain-of-function mice also have exacerbated infarct sizes and vessel permeability after experimental stroke. These effects were rescued by activation of Tie2 signaling using a vascular endothelial protein tyrosine phosphatase inhibitor [125]. These studies highlight the notion that pericyte function is central in regulating BBB/BRB function. However, the factors made by pericytes that promote the BBB and BRB remain unknown.

#### Glia

In addition to pericytes, glial cells also contribute to the regulation of the NVU and BBB/BRB. Recent research has clarified critical signaling pathways from astrocytes that promote barrier properties. The most prevalent glial cells in the brain and retina are astrocytes as well as Müller cells in the retina. Due to their organization and spatial arrangement, glial cells are considered to be the bridge between the neurons and the vasculature, providing immunosurveillance, nutritional and regulatory support [126]. Astrocyte interaction with ECs in brain and retina may be distinct. At the BBB, astrocytes are generally thought to aid in the maintenance of BBB rather than its induction, since astrocytes initially appear at the NVU postnatally [127]. In the retina, astrocytes are present at an early stage, serving as a meshwork template for growing vessels and guiding endothelial cell migration [128, 129]. The majority of retinal astrocytes are limited to the inner vascular plexus, while Müller cells, a specialized retinal glial cell, spans the retina and interacts with all vascular plexus taking over astrocyte functions in the deeper vascular plexus [130–132].

Early experiments showed that transplantation of astrocytes could induce peripheral ECs to form non-leaky vessels [107] implying that glial cells express molecules that regulate barrier properties in ECs. In addition to Wnts, which will be discussed in further detail in the next section, several other astrocyte-derived signals have been identified that regulate BBB function, including sonic hedgehog, angiopoietins, angiotensin, and ApoE.

Sonic hedgehog (Shh) signaling is involved in numerous developmental processes, including neuronal differentiation, axon guidance, and angiogenesis [133]. Shh is produced by astrocytes and has also been identified as important for BBB formation. In physiological conditions, Shh binds and inhibits the EC receptor patched (Ptch) causing de-repression of the co-receptor smoothened (Smo) and consequent activation of the Gli family of transcription factors [134]. Shh knockout mice are embryonic lethal and present abnormalities in BBB formation, having decreased expression of TJ proteins occludin and claudin-5 [135]. Conditional deletion of Smo in ECs revealed that this decrease in TJs protein expression was correlated with vessel leakage, indicating a requirement for Shh signaling in BBB formation during embryonic development.

Astrocytes are also known to secrete angiotensinogen [136], a precursor of angiotensin II that signals through type 1 Ang receptor (AT1) in ECs. Activation of AT1 was shown to restrict ECs permeability through recruitment of occludin to lipid raft membrane microdomains. Accordingly, angiotensinogen deficient mice exhibit a leaky BBB with disorganized occludin strands [137].

Additionally, astrocytes are one of the main sources of ECM, critical in maintaining BBB function. Astrocyte-specific deletion of laminin induces pericyte dysfunction, loss of astrocyte end-feet coverage and BBB leakage associated with decreased expression of occludin and claudin-5 [138, 139], supporting the concept that astrocytes are important in the maintenance of BBB/NVU integrity.

#### Wnt/Norrin-beta-catenin signaling

The Wnt/beta-catenin signaling pathway has been identified as a unique CNS angiogenic program that also induces barrier properties in ECs [140–142]. Wnt molecules are secreted by neural progenitors and glial cells to induce signaling in ECs required for BBB/BRB formation.

Different Wnt signaling pathways are activated when Wnt proteins connect with high affinity to Frizzled (Fzd) receptors. The canonical Wnt signaling pathway causes beta-catenin to stabilize and accumulate in the nucleus, where it binds to transcription factors from the lymphoid enhancer factor/T cell factor (Lef/Tcf) family to induce the transcription of target genes [143].

Endothelial targeted beta-catenin gene deletion leads to specific CNS angiogenesis defects and loss of BBB properties. Besides vascular malformation, these mice show decreased expression of GLUT1, reduced border staining of claudin-5, and upregulation of PLVAP, leading to increased paracellular and transcellular BBB permeability [140–142]. Liebner and colleagues also showed that canonical Wnt signaling is active in ECs during brain angiogenesis and becomes progressively downregulated during vessel maturation, and that inducing beta-catenin signaling earlier is sufficient to accelerate BBB maturation [141]. Targeting astrocytic release of Wnt molecules by conditionally knocking out the Wnt secretion mediator evenness interrupted (Evi) gene leads to brain edema, increased vascular permeability and decreased coverage of brain capillaries by astrocytic end-feet, highlighting the importance of Wnt signaling from astrocytes to EC and barrier maintenance [144].

In mammals, 19 Wnt proteins have been identified and ten different Frizzled receptors have been discovered. Throughout the CNS, Wnt ligands, Frizzled receptors and co-receptors are differentially expressed, with Wnt1, Wnt3, Wnt3a, Wnt4, and Wnt7a/b having a role in BBB formation [140, 142]. Regarding Frizzled receptors, CNS ECs preferentially express Fzd4, Fzd6 and Fzd8 [140].

**Fig. 2:**
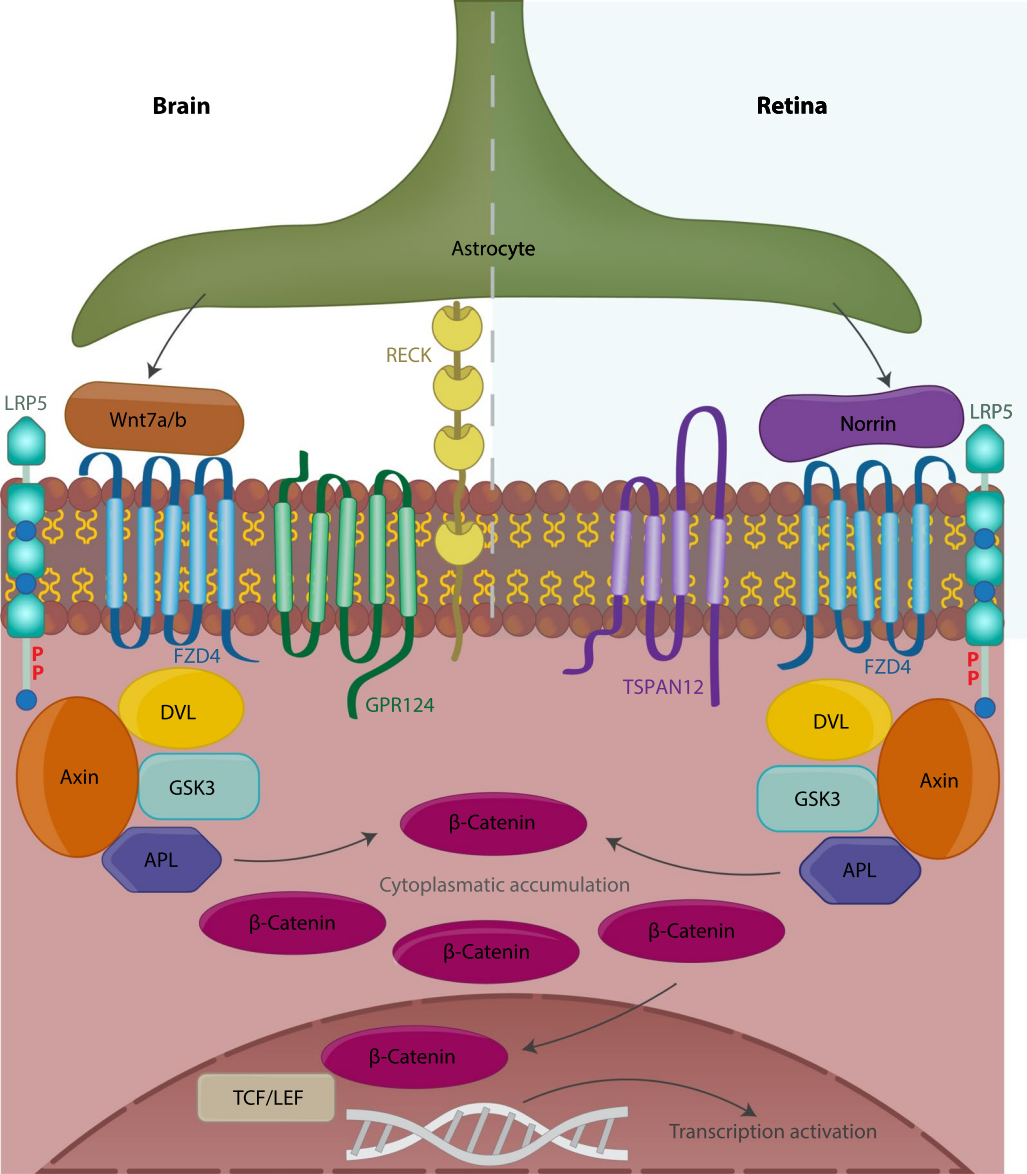
Beta-catenin signaling in CNS ECs. In the brain Wnt7a/b binds specifically to the receptor complex Fzd4/Lrp5/Gpr124/Reck (left) and in the retina norrin binds specifically to Fzd4/Lrp5/Tspan12. Upon ligand binding the canonical Wnt signaling promotes the accumulation of beta-catenin in the cytoplasm and its translocation to the nucleus where it binds to TCF/LEF to promote the transcription of target genes that include genes promoting the induction and maintenance of the barrier

Wnt7a and Wnt7b have been identified as the dominant ligands in the brain cortex [140, 142] and mice lacking both Wnt7a and Wnt7b are embryonic lethal, presenting reduced angiogenesis, abnormal vascular structures and defects in BBB formation [142]. Genetic deletion of GPR124, an orphan member of the G protein-coupled receptor family expressed in ECs and pericytes revealed that these mice presented a similar phenotype to mice lacking Wnt7a and Wnt7b, displaying CNS vascular abnormalities including reduced vascular sprouting, hemorrhages, and also presenting increased Glut1 and PLVAP expression [145–147]. Endothelial cell specific deletion of GPR124 revealed that the effects observed in the global knockout were due to decreased signaling in ECs [147]. GPR124, together with RECK (a GPI-anchored protein) were later identified as coactivators of Wnt7a and 7b signaling through Fzd4 and LRP5/6, enhancing beta-catenin signaling dramatically [148–150]. Reck endothelial-specific knockout mice present a similar phenotype to EC-specific GPR124 knockout and both can be rescued by beta-catenin gain-of-function [151]. Further mechanistic insight revealed that the potentiation of Wnt signaling by GPR124 relies on dishevelled, a critical component of Wnt signaling [152]. Dishevelled recruits Gpr124 and the associated Reck-bound Wnt7 into dynamic Wnt/Reck/Frizzled/Lrp5/6 signalosomes, thus concentrating ligand availability for Fzd signaling [153]. This complex seems to contribute an essential role in brain-specific angiogenesis and BBB formation (Fig. 2).

Interestingly, BRB formation requires a different, but also specific ligand/receptor complex. In the retina, Norrin, a TGF-beta family molecule, binds with high affinity to Fzd4, [154], dependent on the presence of co-receptor TSPAN12 [155] and together with LRP5/6, strongly promotes beta-catenin signaling (Fig. 2) [155–157]. In humans, mutations in any of these genes lead to retinal diseases including the Norrie disease and familial exudative vitreoretinopathies (FEVR), characterized by severe hypovascularization, hemorrhages and blindness [158]. These phenotypes have been recapitulated in knockout mice for Norrin (Ndp), Fzd4, LRP5 and Tspan12. These mice all lack the deep capillary beds and exhibit high vascular permeability, revealing that this signaling complex is required for both retinal angiogenesis and BRB formation [97, 155, 156, 159]. Gene deletion of either Ndp or Fzd4 showed that the increase in vascular permeability was correlated with a reduction in claudin-5 at the cell border and an increase in PLVAP, suggesting that this signaling is required to restrict both the transcellular and paracellular pathways [159]. Importantly, these phenotypes could be rescued by expressing stabilized beta-catenin, demonstrating the requirement of canonical Wnt signaling for retinal barriergenesis. Recently, it was proposed that norrin signaling also plays a role in limiting BRB transcytosis through direct transcriptional activation of Mfsd2a. LRP5 and Ndp knockout mice present high retinal vascular permeability with increased levels of caveolin-1 induced transcytosis together with low levels of Mfsd2a, a known repressor of caveolin-1 mediated transcytosis. Over expression of Mfsd2a in Lrp5 knockout retinas was able to restore transcytosis levels back to normal [160].

Wnt/beta-catenin signaling has also been shown to be necessary for BBB/BRB integrity in mature ECs. Adult mice with Fzd4 deletion or anti-Frizzled 4 blocking antibody showed similar alterations to those observed in Ndp and Fzd4 developmental knockout, with high PLVAP expression and permeability of the BBB/BRB [159, 161]. Additionally, disruption of endothelial-cell specific beta-catenin alone induced BBB breakdown and downregulation of the TJs proteins claudin-1 and -3 in adult mice [162]. Conversely, beta-catenin gain-of-function in FEVR models is sufficient to restore BRB integrity postnatally [141, 149, 159]. Therefore, beta-catenin signaling is required not only for the induction of BRB and BBB characteristics during development, but also for barrier maintenance.

Importantly, adult BBB maintenance requires a continuous and low beta-catenin signal [162]. Recently, Sox17, a known angiogenesis inducer was identified as an important signaling molecule in regulating beta-catenin activity in adult brain ECs. Sox17 is also required for BBB formation and acts in concert with Wnt/beta-catenin signaling. Beta-catenin signaling increases Sox17 that, in turn, maintains the correct beta-catenin activity after vascular development [163]. Further, additional studies have shown that prolonged high levels of beta-catenin signaling lead to alterations in vascular stability [97, 164, 165] including alpha-catenin mutations in humans leading to overactivation of beta-catenin and a FEVR phenotype [165]. Together, these data indicate that Wnt/beta-catenin signaling is required for BBB/BRB formation and maintenance and that beta-catenin must be appropriately regulated for barrier maintenance.

### BBB and neural function

**Fig. 3:**
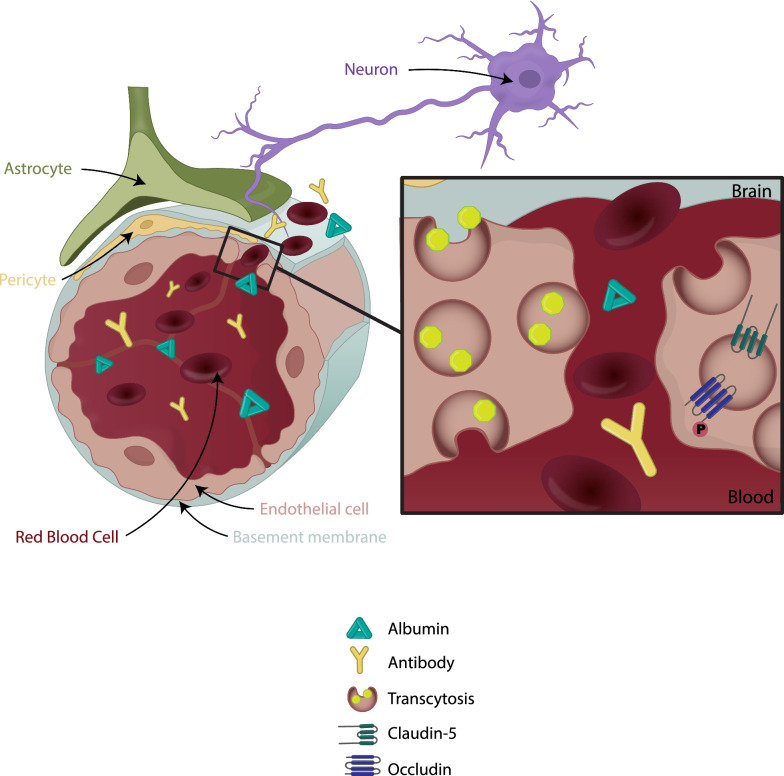
Neurovascular unit breakdown. In several disease states, such as stroke or diabetic retinopathy, a number of factors contribute to BBB breakdown and both paracellular and transcellular transport are increased. Loss of pericytes and gliosis further exacerbate the increase in permeability leading to infiltration of harmful substances to neurons and causing neuronal dysfunction. The process of barrier loss and neural degeneration is hypothesized to lead to a feed-forward pathology extending the disease process. Methods that target barrier breakdown may halt this process

Multiple reviews have cited the requirement of a functional and intact BBB/BRB to maintain the microenvironment required for proper neural signaling and numerous studies correlate breakdown of BBB integrity with neurological dysfunction (Fig. 3). For example, expression of claudin-5 is decreased in the brains of depressed patients, and it positively correlates with resilience to social stress in mice [166] and in a mouse model of depression, transient reduction of claudin-5 levels with siRNA exacerbates depression-like behaviors, while chronic antidepressant treatment rescued claudin-5 loss and promoted resilience [166]. Similarly, claudin-5 protein levels are significantly decreased in surgically resected brain tissue from patients with treatment-resistant epilepsy who present widespread BBB disruption by MRI [167]. In mice, targeted disruption of claudin-5 in the hippocampus exacerbates the excitotoxin kainic acid-induced seizures and BBB disruption, while upregulating claudin-5 expression was sufficient to reduce kainic acid-evoked seizures [167]. Alzheimer’s disease has also been associated with changes in BBB permeability, but the relationship is not simple (reviewed in [168]). However, only recently has modern genetic approaches been used to elucidate precisely how loss of the BBB/BRB impacts neural function or how restoration of the BBB may prevent or restore loss of neural function. For example, claudin-5 gene deletion in the adult mouse brain was shown to be sufficient to induce a psychosis like-behavioral phenotype, with impairments in learning and memory, anxiety-like behavior and sensorimotor gating [169]. Persistent suppression of claudin-5 in these mice led to the development of seizures and 100% rate mortality within 40 days [167, 169]. These studies both emphasize the crucial role of functional TJs in normal neurological function but also demonstrate that loss of barrier properties may be sufficient to induce disease phenotype.

Recent studies have also highlighted the importance of maintaining vascular endothelial TJs in preserving visual function in models of diabetic retinopathy. In retinal ECs, occludin phosphorylation at S490 [170] promotes its ubiquitination and internalization leading to TJ disassembly and vascular permeability [171–173]. Endothelial specific expression of the non-phosphorylatable alanine mutant (S490A) is able to prevent diabetes-induced loss of BRB integrity by preventing TJ disassembly [174]. Importantly, prevention of diabetes-induced permeability resulted in preservation of visual function, as measured by visual acuity and contrast sensitivity, highlighting the importance of early protection of the BRB in diabetes and the relationship of the barrier to visual function [174]. A second study has demonstrated the role of occludin phosphorylation at S490 as a major regulator of tissue plasminogen activator (tPA)-induced BBB permeability in the context of stroke [175]. Preventing occludin phosphorylation either genetically using vascular endothelial specific expression of the stable, S490A non-phosphorylatable form of occludin, or by inhibiting PKCbeta, blocked stroke-induced permeability while decreasing stroke volume and improving functional outcome. Importantly, blocking the increase in BBB permeability with the S490A expression in the vascular endothelium or by providing PKCbeta inhibitor 5 h after stroke, was also able to prevent intracerebral hemorrhage induced by late-thrombolytic therapy [175]. Late tPA induced hemorrhage is associated with high mortality [176] and PKCbeta treatment may provide a means to allow late tPA thrombolytic activity without barrier breakdown.

Others have demonstrated the value of preserving the BBB in stroke. Vascular endothelial conditional expression of a constitutively active mutant form of actin depolymerizing factor (ADFm) or cofilin, reducing actin polymerization leads to reduced permeability after stroke and reduced TJ disassembly at endothelial cell border [177]. Importantly, preserving the BBB significantly improved neurological outcome in the stroke mice [177]. A second study using vascular endothelial conditional expression of heat shock protein 27 (HSP27), which also prevents actin polymerization, also demonstrated reduced BBB permeability and improved neurologic outcome after stroke [178].

Analysis of poststroke human and mouse blood microvessels have identified claudin-1 as highly expressed in leaky brain microvessels [26]. This newly synthesized claudin-1 was shown to be incorporated into the TJ complex with established interaction with ZO-1 and building homophilic cis- and trans-interactions [26]. Claudin-5 strands, both homophilic cis- and trans-interactions, and the claudin-5/ZO-1 interaction were reduced when claudin-1 was introduced into the TJ complex. This altered claudin-5 incorporation into the TJ complex prevents BBB recovery causing BBB permeability. Using a specific peptide that targets claudin-1 reduced BBB permeability after stroke and consequently improved neurological recovery [26].

Recent studies have also targeted microRNAs that regulate TJs expression. Specifically, miR-15a/16-1 cluster directly binds to claudin-5 3′UTR, decreasing its expression and endothelial cell-specific deletion of this cluster in mice was able to increase claudin-5 expression, leading to improvement of BBB dysfunction and post-stroke functional outcome [179]. Additionally, miR-501-3p was shown to directly bind to the 3′UTR region of ZO-1 to decrease its expression [180]. Administration of a locked nucleic acid-modified antisense oligonucleotide targeting miR-501-3p was able to rescue ZO-1 expression and BBB disruption in a mouse model of vascular cognitive impairment, significantly improving working memory deficits [180].

Considering that barrier function also regulates the exchange of nutrients and waste with the parenchyma, a reduction in permeability may also have a detrimental impact on neuronal activity.

Inducing beta-catenin signaling in the leaky vessels of the circumventricular organs converts them into BBB-like vessels by increasing claudin5 and decreasing PLVAP expression, overall reducing vascular permeability [98]. Endothelial tightness was accompanied by an increase in neuronal activity in that region; however, this non-physiological increase presented detrimental effects in basic behaviors [98].

In Alzheimer's disease (AD), which is characterized by the accumulation of neurotoxic amyloid-β (Aβ) peptides leading to a gradual decline in cognitive function [181], the impairment of BBB ability to properly promote Aβ clearance has been shown to have pathological effects on neuronal function and contribute to AD progression. Reduced levels of glucose transporter GLUT1 were reported in brain microvessels of AD patients and murine models [89]. Transgenic mice overexpressing human amyloid β-peptide showed accelerated Aβ accumulation in the brain when Glut1 was conditionally deleted from ECs [89]. These mice presented increased progressive neuronal dysfunction, behavioral deficits, neuronal loss and neurodegeneration [89]. GLUT1 deficiency effects in reducing Aβ-clearance and accelerating AD pathology were due to consequent reduced expression of LRP1, a key Aβ clearance transporter, in the microvasculature [89]. LRP1-mediated transcytosis has been shown to contribute to Aβ-clearance [182]. Endothelial-specific deletion of Lrp1 showed reduced brain efflux of injected Aβ-peptides in wild-type mice. In a transgenic mouse model of AD, this conditional deletion of Lrp1 lead to a reduction of plasma Aβ levels and elevated soluble brain Aβ, resulting in a decline of spatial learning and memory deficits [182]. In a separate study, blocking Lrp1 expression with antisense mRNA resulted in similar accumulation of radiolabeled Aβ-peptides in the brain and impaired learning and memory [183]. Targeting restoration of LRP1 or GLUT1 expression may lead to increased LRP1-mediated Aβ clearance, representing a novel strategy for AD therapy. Indeed, additional efflux transporters such as P-glycoprotein may also be targets to promote Aβ clearance [184].

Recent studies have shown that targeting the paracellular route might also enhance Aβ clearance. In a murine model of familial AD suppression of the TJ proteins claudin-5 and occludin by systemic siRNA allowed for diffusion of soluble human Aβ monomers from the brain to the blood [185]. These animals presented increased plasma levels of Aβ monomers and reduced brains levels of neurotoxic Aβ with a concomitant improvement in cognitive function [185]. Importantly, the authors reported that siRNA to claudin-5 and occludin did not induce changes in BBB permeability to large plasma proteins such as immunoglobulins that could have a detrimental effect.

Targeting paracellular permeability has also been tested in the context of cerebral edema and brain swelling, which are associated with higher risk of permanent brain damage and mortality. Administration of siRNA against claudin-5 post induction of a cold-induced cerebral edema allowed for enhanced exchange of accumulated extra-neural water, decreasing brain-swelling, and improving the cognitive outcome after the focal injury [186]. The increase in BBB permeability was achieved in a temporary and size-selective manner [186]. A similar approach for the use of siRNA targeting claudin-5 for clearance of edema from the optic nerve head by intravitreal injection has been proposed by the same group [187] but has yet to be tested. Collectively, these studies emphasize the importance of the BRB and BBB and demonstrate that targeting endothelial vascular permeability can improve neurologic outcome.

## Conclusion

Transgenic approaches have proven to be highly valuable in understanding the complex multi-cellular interaction necessary for formation of the BBB and BRB and have provided critical insight into required factors for proper barriergenesis. The use of transgenic mice has demonstrated the importance of the BBB and BRB for proper neural function in a physiological setting. These studies have also begun to elucidate how manipulation of the barrier, either by promoting tightening of the barrier or permeability, may provide important therapeutic options to address diseases of the CNS. Future studies using stem cells and organ culture systems may provide critical information on the factors sufficient to promote formation of the BBB and provide new avenues to quickly address fundamental questions regarding the NVU. However, the use of transgenic animals in the study of the BBB and BRB promises to continue to provide important physiological insight and novel understanding of the NVU.

## Data Availability

Not applicable.
